# Adverse Effects of Fenofibrate in Mice Deficient in the Protein Quality Control Regulator, CHIP

**DOI:** 10.3390/jcdd5030043

**Published:** 2018-08-15

**Authors:** Saranya Ravi, Traci L. Parry, Monte S. Willis, Pamela Lockyer, Cam Patterson, James R. Bain, Robert D. Stevens, Olga R. Ilkayeva, Christopher B. Newgard, Jonathan C. Schisler

**Affiliations:** 1 McAllister Heart Institute at The University of North Carolina at Chapel Hill, Chapel Hill, NC 27599, USA; saranya_ravi@med.unc.edu (S.R.); tlparry@iu.edu (T.L.P.); plockyer9@gmail.com (P.L.); 2 Indiana Center for Musculoskeletal Health, University of Indiana School of Medicine, Indianapolis, IN 46202, USA; willisms@iu.edu; 3 The Office of the Chancellor, University of Arkansas for Medical Sciences, Little Rock, AR 72205, USA; CPatters@uams.edu; 4 Sarah W. Stedman Nutrition and Metabolism Center and Duke Molecular Physiology Institute, Departments of Pharmacology and Cancer Biology and Medicine, Duke University Medical Center, Durham, NC 27701, USA; james.bain@duke.edu (J.R.B.); steve018@mc.duke.edu (R.D.S.); olga.ilkayeva@duke.edu (O.R.I.); newga002@mc.duke.edu (C.B.N.); 5 Department of Pharmacology and Department of Pathology and Lab Medicine, The University of North Carolina at Chapel Hill, Chapel Hill, NC 27599, USA

**Keywords:** metabolism, fibrates, fibrosis, metabolomics, pressure overload, autophagy, mitophagy

## Abstract

We previously reported how the loss of CHIP expression (Carboxyl terminus of Hsc70-Interacting Protein) during pressure overload resulted in robust cardiac dysfunction, which was accompanied by a failure to maintain ATP levels in the face of increased energy demand. In this study, we analyzed the cardiac metabolome after seven days of pressure overload and found an increase in long-chain and medium-chain fatty acid metabolites in wild-type hearts. This response was attenuated in mice that lack expression of CHIP (*CHIP*^−/−^). These findings suggest that CHIP may play an essential role in regulating oxidative metabolism pathways that are regulated, in part, by the nuclear receptor PPARα (Peroxisome Proliferator-Activated Receptor alpha). Next, we challenged *CHIP*^−/−^ mice with the PPARα agonist called fenofibrate. We found that treating *CHIP*^−/−^ mice with fenofibrate for five weeks under non-pressure overload conditions resulted in decreased skeletal muscle mass, compared to wild-type mice, and a marked increase in cardiac fibrosis accompanied by a decrease in cardiac function. Fenofibrate resulted in decreased mitochondrial cristae density in *CHIP*^−/−^ hearts as well as decreased expression of genes involved in the initiation of autophagy and mitophagy, which suggests that a metabolic challenge, in the absence of CHIP expression, impacts pathways that contribute to mitochondrial quality control. In conclusion, in the absence of functional CHIP expression, fenofibrate results in unexpected skeletal muscle and cardiac pathologies. These findings are particularly relevant to patients harboring loss-of-function mutations in CHIP and are consistent with a prominent role for CHIP in regulating cardiac metabolism.

## 1. Introduction

Carboxyl terminus of Hsc70-interacting protein (CHIP, encoded by the gene *STUB1*) is a dual-function enzyme, which has both chaperone-related and ubiquitin ligase activities [1]. Coding mutations in *STUB1* cause a rare multi-organ disease, and is now identified as SCAR16 (autosomal recessive spinocerebellar ataxia 16, OMIM: 615768). Clinical phenotypes of SCAR16 patients include cerebellar ataxia, cognitive dysfunction, and, in some cases, hypogonadism [2,3]. Moreover, studies suggest that the majority of CHIP coding mutations result in protein destabilization and loss-of-function [4,5]. The impact of CHIP mutations on cardiac function in SCAR16 patients has not been reported. However, several laboratories including our own, demonstrated a cardio-protective role for CHIP in mouse models including cardiac hypertrophy and cardiac ischemia-reperfusion injury [6]. For example, CHIP contributed to cardio-protection by preventing cardiomyocyte apoptosis after ischemia [7,8,9]. Additionally, over-expression of CHIP prevented cardiac fibrosis and inflammation in an angiotensin II-induced model of hypertension [10] and prevented cardiac myogenesis and pathological hypertrophy under conditions of hyperinsulinemia [11]. In contrast, the genetic deletion of CHIP was found to enhance both physiological and pathological hypertrophy [12,13]. During pressure overload, the loss of CHIP also decreased survival, compromised cardiac function, and reduced metabolic reserves [13]. Metabolic insufficiency in *CHIP*^−/−^ mice following cardiac pressure overload occurred in part due to the inability to increase the activation of 5′ AMP-activated protein kinase (AMPK) [13]. CHIP is required for LKB1-mediated phosphorylation and activation of AMPK through conformational changes to AMPK in the presence of CHIP that ultimately increase AMPK activity [13]. AMPK is a master metabolic regulator that senses the cellular energy status through the binding of adenine nucleotides. Increasing AMPK activity promotes fatty-acid and glucose oxidation. These data suggest that targeting other metabolic regulators besides AMPK could potentially blunt the metabolic dysfunction observed in *CHIP*^−/−^ hearts.

One pharmacological approach to promote cardio-protection in metabolically impaired hearts is the use of fibrates, which is a class of pharmaceuticals commonly used to lower serum triglycerides and increase HDL levels in patients with hyperlipidemia [14] as well as for secondary prevention of cardiovascular disease and stroke [15]. Fibrates are agonists for Peroxisome Proliferator-Activated Receptor alpha (PPARα), which is a nuclear receptor protein that is activated by endogenous ligands such as free fatty acids [14]. Upon activation, PPARα induces the hepatic expression of genes involved in fatty acid oxidation, cellular uptake of fatty acids, synthesis of high-density lipoproteins (HDL), apoproteins, and lipoprotein lipase while suppressing the expression of apolipoprotein C-III [16,17,18,19]. In this paper, we tested the pleiotropic effects of fenofibrate, which has been in use since 1975 and is one of the most commonly prescribed fibrates [20], on cardiac function in *CHIP*^−/−^ mice with the initial goal of using fibrates to possibly compensate for the metabolic deficiencies that occur when the heart is challenged with a chronic pressure overload. However, in our pharmacological testing of fenofibrate on cardiac function in non-stressed *CHIP*^−/−^ mice, we observed unexpected detrimental effects in skeletal muscle and heart including decreased cardiac function and increased myocardial fibrosis. Moreover, we found abnormal mitochondria in cardiac sarcomeres in *CHIP*^−/−^ mice treated with fenofibrate as well as decreased expression of genes involved in autophagy and mitophagy. Our data suggest that CHIP expression is necessary for the pleiotropic effects of fenofibrate on cardiac function.

## 2. Materials and Methods

### 2.1. Animals

We used CHIP^+/−^ breeding pairs on a 129SvEv background (129S(B6)-*Stub1^tm^*^1*Cpat*^/Mmnc) to generate wild-type (*CHIP*^+/+^) and *CHIP*^−/−^ mice. All animal work was performed according to the Guide for the Care and Use of Laboratory Animals under approved IACUC animal use protocols within the AAALAC accredited program at The University of North Carolina at Chapel Hill (Animal Welfare Assurance Number A-3410–01) that comply with NIH standards for care and use.

### 2.2. Metabolomic Analyses of Hearts

Amino acids, acylcarnitines, and organic acids were measured in snap-frozen, powdered mouse heart tissue via stable isotope dilution techniques [21,22,23] (N = 3 hearts per genotype, per condition). Samples were equilibrated with a cocktail of internal standards and de-proteinated by precipitation with methanol. Aliquots of the supernatants were dried and then esterified with hot, acidic methanol (acyl-carnitines) or n-butanol (amino acids). Data were acquired using a Waters Acquity^TM^ UPLC system equipped with a TQ (triple quadrupole) detector and a data system controlled by the MassLynx 4.1 operating system (Waters, Milford, MA, USA) [21,22]. We quantified the concentration of organic acids in samples using the Trace Ultra GC coupled to ISQ MS operating under Xcalibur 2.2 (Thermo Fisher Scientific, Inc., Waltham, MA, USA) [23].

Metabolite concentrations were analyzed with Metaboanalyst (v3.0) run in the statistical package R (v3.03) [24,25]. Features with missing data greater than 50% were removed and the remaining missing values were estimated using KNN (K nearest neighbor). Data were log-transformed and mean-centered. All metabolites were first evaluated using principal component analysis (PCA). Next, differences in metabolite concentrations were determined using two-way ANOVA with genotype and surgery as the main effects. Metabolites were considered differentially present using a false discovery rate cut off of <10% at either the main effect level or at the interaction level. Raw, processed, and normalized data along with ANOVA results are available as Supplementary Material. Semi-supervised hierarchical clustering (Euclidean distance and Ward clustering algorithm) was performed on the differential variables and visualized using a heatmap.

### 2.3. Ex Vivo Oxidation and ATP Assays

Oxidation studies utilized fresh heart tissue homogenized in oxidation buffer (75 mM Tris-HCl, pH 7.4) containing 25 mM sucrose, 30 mM KCl, 5 mM MgCl_2_, 10 mM KPO_4_, 1 mM EDTA, 1 mM NAD^+^, 25 µM cytochrome c, 0.1 mM acetyl-CoA, 0.5 mM malate, 0.5 mM L-carnitine, 5 mM ATP [26,27]. Substrate oxidation was measured using [1-^14^C]oleate or [U-^14^C]glucose measured in triplicate per heart sample (N = 3 per genotype, per condition) using approximately 1 mg to 2 mg of heart protein per replicate. Reactions were terminated by adding 100 μL of 70% perchloric acid, trapping ^14^CO_2_ in 200 μL of 1 N NaOH and counted in Uniscint BD scintillation solution (National Diagnostics, Atlanta, GA, USA). Acid-soluble metabolites from oleate oxidation were included in total fatty acid oxidation rates and results are expressed as nanomoles of ATP produced per hour per milligram of protein using 129 and 38 moles of ATP per mole of fatty acid or glucose substrate, respectively [28,29,30]. Cardiac ATP concentrations were measured in fresh heart tissue using the ATP Bioluminescence Assay Kit HS II (Roche, Indianapolis, IN, USA) normalized to protein concentration for each heart sample (N = 6 per genotype, per condition).

### 2.4. Fenofibrate Feeding

Mice, 16–18 weeks-of-age, were randomized to receive either standard mouse chow (Prolab RMH 3000, Purina LabDiet, St. Louis, MO, USA) or standard chow formulated with 0.05% *w*/*w* fenofibrate (F6020, Sigma-Aldrich Corp., St. Louis, MO, USA) [31]. Mouse chow (fenofibrate and standard sham chow) were administered ad libitum starting on day 1 of the protocol and stopped after five weeks. The number of animals and analyses are listed in Table 1.

### 2.5. RNA Isolation and Quantitative Polymerase Chain Reaction (qPCR) Analysis of Gene Expression

Total RNA was isolated from mouse liver or heart (N = 3 animals per genotype, per condition) using the AllPrep DNA/RNA/Protein Mini Kit (Qiagen, Germantown, MD, USA) and 500 ng of RNA was reverse-transcribed into cDNA using iScript Reverse Transcription Supermix (Bio-Rad, Laboratories, Inc., Hercules, CA, USA). Gene expression assays were performed using either Universal Probe (UPL) Assays (Roche) or SYBR green chemistry with the indicated oligonucleotides (Table 2) with FastStart Universal Probe Master Rox (Roche) or FastStart Universal SYBR Green Master Rox (Roche), respectively, on the 7900HT instrument (Applied Biosystems, Foster City, CA, USA). Efficiencies of qPCR reactions (1.9–2.1) were confirmed using serial dilution of pooled samples. Three biological replicates were used in triplicate technical replicates per gene. Relative mRNA levels were calculated using the delta Cq method. The data was centered using the geometric mean of all control chow samples and 18S ribosomal RNA levels (4310893E, Applied Biosystems) to normalize loading.

### 2.6. Measurement of Blood Chemistry

After the animals were euthanized, blood was collected in tubes containing EDTA/citrate. The blood was then centrifuged at 3000× *g* for three minutes to separate the plasma from the red blood cells. Levels of triglyceride, total cholesterol, high-density lipoprotein (HDL) cholesterol, glucose, creatine kinase, and creatine kinase-MB were measured by the Animal Histopathology and Lab Medicine Core at The University of North Carolina at Chapel Hill. Low-density lipoprotein (LDL) cholesterol was calculated using the formula: total cholesterol—HDL—(triglycerides/5).

### 2.7. Echocardiography

The mice underwent conscious echocardiography using the Vevo 770 ultrasound micro-imaging system (VisualSonics, Inc., Toronto, ON, Canada) using the model 707B scan head (30 MHz) [32,33,34]. Two-dimensional guided M-mode echocardiography was performed in the parasternal long-axis view at the level of the papillary muscle. Wall thickness was then determined by measurements of epicardial to endocardial leading edges.

### 2.8. Morphological Analysis of Tissue by Histology and Transmission Electron Microscopy

For histological assessment, mouse hearts (N = 3 hearts per genotype, per condition) were perfused with 4% paraformaldehyde, embedded in paraffin, and sectioned into five-micron sections. Heart sections were stained with either H&E or Masson’s trichrome staining. Whole slides were imaged using an Aperio Scanscope and analyzed using the Aperio Imagescope software (v10.0.36.1805, Leica Biosystems, Buffalo Grove, IL, USA). Fibrosis was determined using the Positive Pixel Count Algorithm to analyze Masson’s trichrome-stained four-chamber sections, with the hue value = 0.66 (blue) and the hue width = 0.1 (detection threshold above a white background [13,31]. The amount of fibrosis (N = 3 hearts per genotype, per condition) was calculated as the area of fibrotic tissue (blue = collagen) as a percentage of total tissue area (above background, white). Each heart was represented by multiple slides including three to four sections per slide. The average fibrosis percentage per heart represents the mean across slides. The myocyte area was determined similarly using the same heart sections using NIH ImageJ (v1.38) based on photomicrographs of a standard graticule ruler. Hearts apices and skeletal muscle were fixed and imaged using a EM910 transmission electron microscope (Zeiss, Thornwood, NY, USA) [35]. 

## 3. Results

### 3.1. Role of CHIP in Fatty Acid Metabolism

**Figure 1:**
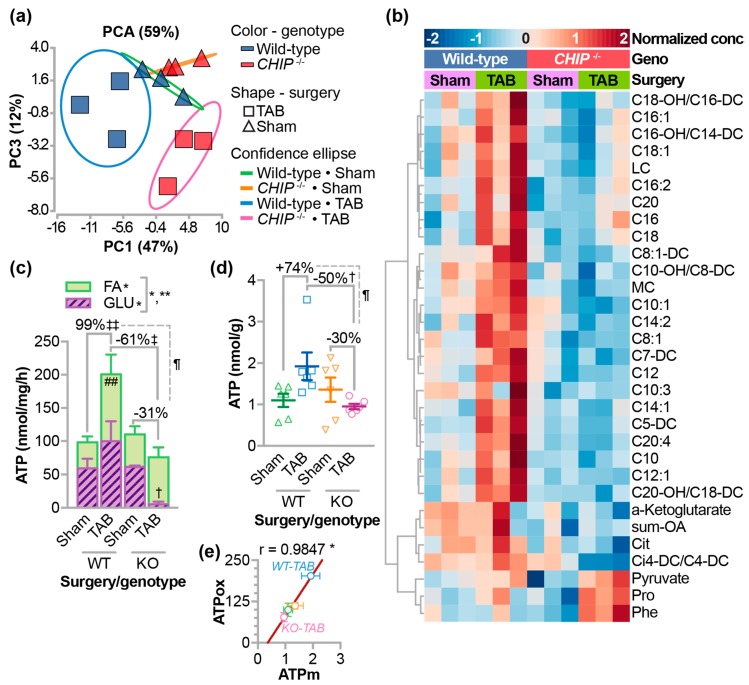
The effects of pressure overload on metabolism in *CHIP*^−/−^ hearts. Metabolomic analysis of whole hearts isolated from wild-type or *CHIP*^−/−^ mice one week after a sham surgery or trans-aortic banding (TAB) were analyzed using (**a**) principal component analysis (PCA) and (**b**) two-way ANOVA, N = 3 hearts per genotype per condition. Variances captured by the first and third principal components (PC) are shown. Differential metabolites via ANOVA (FDR < 10%) were clustered and represented by a heatmap. (**c**) Ex-vivo oxidative ATP generation rates in mouse heart homogenates summarized by the mean ± SEM using either fatty acid (open bars) or glucose (hashed bars) as a substrate. Two-way ANOVA for glucose oxidation, N = 3 hearts per genotype per condition: * *p* < 0.05 of genotype main effect and interaction between genotype and surgery, post-test: ^†^ *p* < 0.05 glucose oxidation in wild-type vs. *CHIP*^−/−^ after one week of TAB. Two-way ANOVA for fatty acid oxidation, * *p* < 0.05 on surgery main effect. Two-way ANOVA for total ATP, * *p* < 0.05 on surgery main effect, ** *p* < 0.01 on genotype main effect and interaction, post-test: ^‡‡^ *p* < 0.01 total ATP in wild-type sham vs. TAB mice, post-test, ^‡^ *p* < 0.05 total ATP in wild-type vs. *CHIP*^−/−^ after one week of TAB and the surgery-dependent percent change in ATP production rates (TAB vs. sham) in wild-type vs. *CHIP*^−/−^ was significant at *p* = 0.039. (**d**) Steady-state ATP levels in mouse hearts represented by dot plot and summarized by the mean ± SEM, N = 6 hearts per genotype, per condition. Two-way ANOVA interaction of the main effects *p* = 0.0177, post-test: ^†^ *p* < 0.05 in wild-type vs. *CHIP*^−/−^ at one week of TAB and the surgery-dependent percent change in ATP levels (TAB vs. sham) in wild-type vs. *CHIP*^−/−^ was significant at *p* = 0.034. (**e**) Pearson correlation analysis of ATP determined by *ex vivo* oxidization rates (ATPox) or measured steady-state ATP levels (ATPm) * *p* = 0.0153.

We previously reported surprisingly low cardiac AMPK activity after one week of pressure overload in *CHIP*^−/−^ mice despite the increase in metabolic demand [13]. AMPK can drive oxidative metabolism including fatty acid and glucose oxidation. We measured differences in metabolites from wild-type and *CHIP*^−/−^ hearts in the context of pressure overload by trans-aortic banding (TAB) for one week [13]. Principal components analysis of 74 acylcarnitine, amino acid, and organic acid metabolites revealed distinct differences in the TAB conditions comparing wild-type and *CHIP*^−/−^ hearts (Figure 1a). We identified differential metabolites via two-way ANOVA including an increase in the carnitine esters of several medium-chain (MC) and long-chain (LC) fatty acids as well as α-ketoglutarate (Figure 1b). The majority of the differences we observed were manifest in increased MC and LC acylcarnitines with pressure overload in wild-type hearts, an effect that was attenuated in *CHIP*^−/−^ hearts. These data suggest that pressure overload in wild-type hearts results in specific changes to oxidative metabolic flux that are distinct from those found in *CHIP*^−/−^ hearts. Using fresh cardiac homogenates, we measured oxidative metabolism under these same conditions. As expected, pressure overload resulted in a compensatory increase in ATP production mostly from an increase in fatty acid oxidation (Figure 1c), which is consistent with the metabolite data (Figure 1b). Remarkably, in samples from pressure-overloaded *CHIP*^−/−^ hearts, there was a dramatic drop in glucose oxidation and, unlike wild-type hearts, there was no increase in fatty acid oxidation, which resulted in an overall decrease in oxidative ATP generation. We also measured steady-state ATP levels (Figure 1d) and observed similar patterns to our calculated ATP values (Figure 1e), which highlights the disparity between wild-type and *CHIP*^−/−^ hearts after pressure overload. Therefore, we conducted a pilot study to determine the effect of treating *CHIP*^−/−^ mice with fenofibrate as a possible approach for rescuing the metabolic defect seen during pressure overload.

### 3.2. Fenofibrate-Activated PPARα Target Genes in the Liver

**Figure 2:**
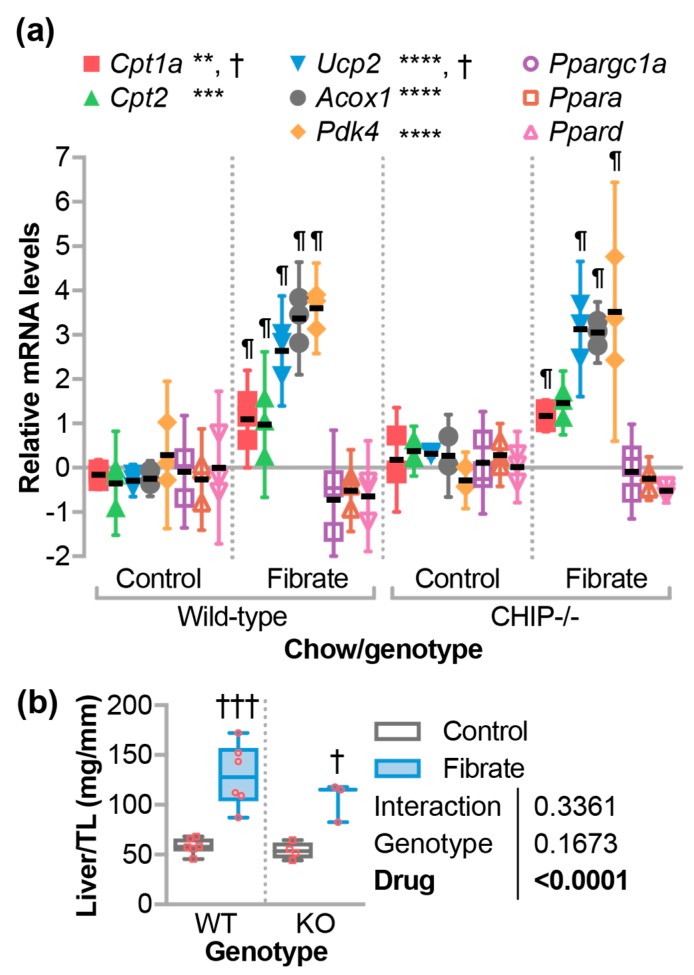
The effect of fenofibrate on liver. (**a**) Quantitative PCR analysis of gene expression in livers from either wild-type or *CHIP*^−/−^ mice fed control or fenofibrate chow, which is represented by dot plot and summarized by the mean ± 95% CI, N = 3 animals per genotype per condition: **, ***, **** indicate *p* < 0.01, 0.001, 0.0001 or ^†^ *p* < 0.05 on the main effect of chow or genotype, respectively, via two-way ANOVA. indicates Tukey’s post-test < 0.05 in comparing fibrate to control conditions within genotypes. (**b**) Liver weight normalized to tibia length (TL) is represented by the boxplot with plotted biological replicates. Results of two-way ANOVA are provided, Tukey’s post-test: ^†^ and ^†††^ indicate *p* < 0.05 and 0.001 comparing control vs. fibrate conditions.

Since the loss of CHIP expression appears to confer a loss of metabolic flexibility, we initiated a study to challenge *CHIP*^−/−^ mice with fenofibrate, which is a PPARα agonist, to determine the effect of a drug known to stimulate oxidative metabolism. Mice were administered fenofibrate by incorporating the drug into the chow (Table 1). To demonstrate that the fenofibrate dose stimulated PPARα activity, we first analyzed known hepatic target genes of PPARα in RNA purified from mouse liver using quantitative PCR (qPCR). As expected, fenofibrate increased expression of *Cpt1a* (Carnitine palmitoyltransferase 1a), *Cpt2* (Carnitine palmitoyltransferase 2), *Ucp2* (Uncoupling protein 2), *Acox1* (Acyl-CoA oxidase 1), and *Pdk4* (Pyruvate dehydrogenase kinase 4) to similar levels in both wild-type and *CHIP*^−/−^ mice (Figure 2a) [36,37,38]. However, there were no changes in *Ppargc1a* (Pparg coactivator 1 alpha), *Ppara*, and *Ppard* expression, which suggests no increases in mitochondrial biogenesis (Figure 2a). Previous reports demonstrated that fenofibrate increased liver weight in mice [38,39] and, in fact, we observed similar increases in liver weight in both wild-type and *CHIP*^−/−^ mice that were fed fenofibrate chow (Figure 2b). Together, these data suggest that the transcriptional and phenotypic responses to fenofibrate in the liver, which is the primary target organ of fibrates, are not affected by the loss of CHIP expression.

### 3.3. Fenofibrate Altered Circulating Cholesterol in Wild-Type But Not CHIP^−/−^ Mice

**Figure 3:**
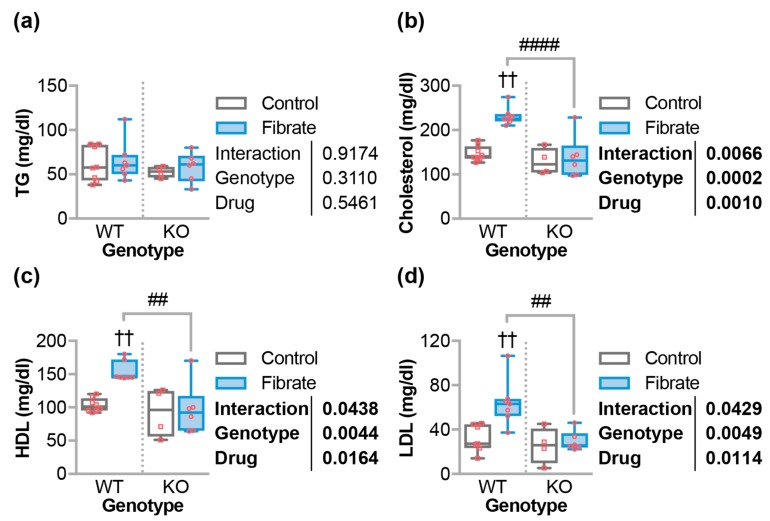
Fenofibrate effects on circulating lipid levels. (**a**) Triglycerides (TG), (**b**) total cholesterol, (**c**) HDL cholesterol, and (**d**) LDL cholesterol in either wild-type (WT) or *CHIP*^−/−^ (KO) mice fed control or fenofibrate chow, which is represented by a boxplot with biological replicates plotted. Results of two-way ANOVA are provided. Tukey’s post-test: ^††^ indicate *p* < 0.01 comparing control vs. fibrate conditions. ^##^ and ^####^ indicate *p* < 0.01 and 0.0001 comparing WT vs. KO conditions.

Fibrates are primarily used to treat hypercholesterolemia and hypertriglyceridemia and, even though we were not studying models of hyperlipidemia, we investigated the impact of fibrates on lipid profiles in our model. As expected, there were no changes in triglyceride levels with respect to genotype or chow (Figure 3a). Paradoxically, we and others observed that fenofibrate increased the levels of total cholesterol in mice on standard chow (non-Western) diets [31,40]. In this study, we observed that fenofibrate led to a 58% increase in the total cholesterol levels in WT mice, which is an effect that was ablated in *CHIP*^−/−^ mice (Figure 3b). The increase in total cholesterol was comprised of similar increases in both high-density lipoprotein cholesterol (HDL-c) and low-density lipoprotein cholesterol (LDL-c) in wild-type mice treated with fenofibrate while these levels did not change in *CHIP*^−/−^ mice (Figure 3c,d).

### 3.4. Differential Effects of Fenofibrate on Skeletal Muscle

**Figure 4:**
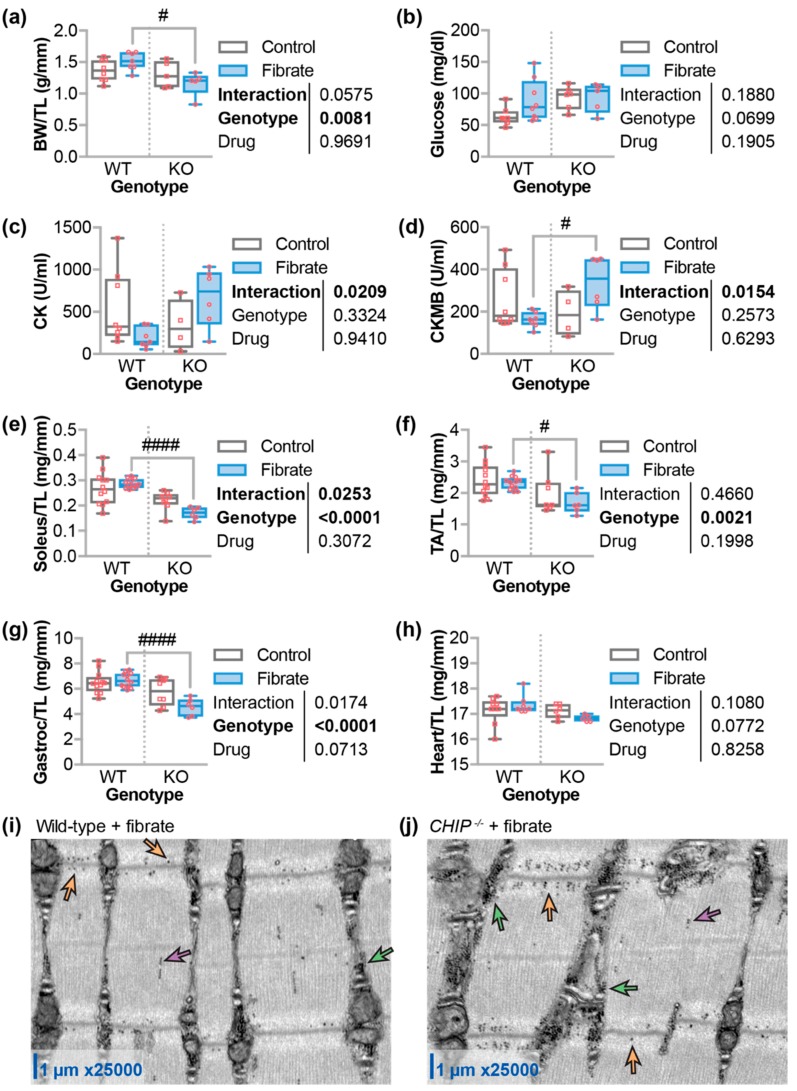
Fenofibrate effects on body weight, glucose levels, and muscle. (**a**) Body weight (BW) normalized by tibia length (TL), circulating levels of (**b**) glucose, (**c**) creatine kinase (CK), or (**d**) creatine kinase MB (CKMB), and weights of (**e**) soleus, (**f**) tibialis anterior, (**g**) gastrocnemius (gastroc), and (**h**) heart, which was normalized by TL, in either wild-type (WT) or *CHIP*^−/−^ (KO) mice that were fed control or fenofibrate chow represented by boxplot with biological replicates plotted. Results of two-way ANOVA are provided. Tukey’s post-test: ^#^ and ^####^ indicate *p* < 0.05 and 0.0001 comparing WT vs. KO conditions. Transmission electron micrographs of gastrocnemius muscle sarcomeres from (**i**) wild-type and (**j**) *CHIP*^−/−^ mice after five weeks of fenofibrate. Glycogen can be seen as small, dense, round objects highlighted by arrows and found in the I-band (orange), A-band (purple), and sarcoplasm (green).

On average, body weights were lower in *CHIP*^−/−^ mice compared to wild-type mice (*p* = 0.0081) and, after five weeks of fenofibrate, *CHIP*^−/−^ mice were 23% lighter than wild-type mice (Figure 4a). *CHIP*^−/−^ mice trended towards higher fasting blood glucose levels compared to wild-type mice. However, fenofibrate did not affect fasting blood glucose in either wild-type or *CHIP*^−/−^ mice (Figure 4b). Given the known defects in *CHIP*^−/−^ skeletal muscle [41], we measured creatine kinase (CK) and creatine kinase-MB (CKMB), which are enzymes that indicate muscle damage. Two-way ANOVA indicated an interaction between the genotype and the drug treatment with higher CK and CKMB levels in *CHIP*^−/−^ mice treated with fenofibrate compared to wild-type mice (Figure 4c,d). This suggests that either skeletal or cardiac muscle may be negatively affected by fenofibrate in the absence of CHIP expression. Therefore, we measured various muscle weights and found that the tibialis anterior, soleus, and gastrocnemius muscles, on average, were reduced in *CHIP*^−/−^ mice compared to wild-type mice treated with fenofibrate (Figure 4e–g). We did not observe any changes in heart weight (Figure 4h), which suggests that the increase in muscle enzymes seen in *CHIP*^−/−^ mice treated with fenofibrate is likely indicative of skeletal muscle atrophy. We previously described accumulation of lamellar bodies in the gastrocnemius muscle in *CHIP*^−/−^ mice. Fenofibrate did not appear to affect the appearance of these structures. However, in both wild-type and *CHIP*^−/−^ mice on fenofibrate chow, we observed glycogen accumulation throughout the sarcomere predominantly in the I-band and the sarcoplasm (Figure 4i,j).

### 3.5. Fenofibrate Decreased Cardiac Function and Increased Fibrosis in CHIP^−/−^ Mice

**Figure 5:**
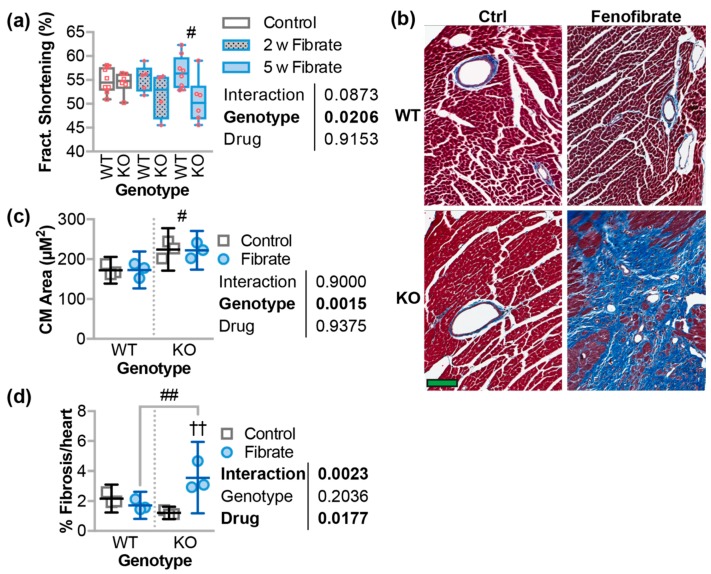
Changes in cardiac function and structure due to fenofibrate. (**a**) Fractional (fract.) shortening over the time course of the study, which is represented by boxplot with biological replicates plotted. Results of two-way ANOVA are provided. Tukey’s post-test: ^#^ *p* < 0.05 comparing wild-type (WT) vs. *CHIP*^−/−^ (KO) conditions. (**b**) Micrographs of Masson’s trichrome staining of heart sections in which the scale bar represents 100 microns. (**c**) Cardiomyocyte area and (**d**) the percentage of fibrotic cardiac tissue represented by dot plot and summarized by the mean ± 95% CI, N = 3 animals per genotype per condition. The results of two-way ANOVA are provided. Tukey’s post-test: ^#^ and ^##^ *p* < 0.05 and 0.01 comparing WT vs. KO conditions; ^††^ *p* < 0.01 control vs. fibrate conditions.

We next measured cardiac function at two and five weeks after fenofibrate treatment using conscious echocardiography (Table 3). There were changes at the two-week time point in *CHIP*^−/−^ mice, which included an increase in interventricular septum size in both diastole and systole, as well as an increase in the calculated left-ventricular (LV) mass, which is suggestive of LV wall thickening. However, these changes were resolved at the five-week time point. Fenofibrate also caused a modest 11% decrease in cardiac function after five weeks, which was measured by fractional shortening (Figure 5a). We analyzed ventricle tissue to determine cardiomyocyte surface area and fibrosis using histochemical approaches developed in our lab (Figure 5b) [13,31]. On average, *CHIP*^−/−^ mice had larger cardiomyocytes, but these parameters were not changed with fenofibrate. In contrast, we observed increased cardiac fibrosis in *CHIP*^−/−^ mice after fenofibrate treatment (Figure 5d). Although this increase in fibrosis is modest compared to what we observed in pressure-overloaded hearts (upwards to 20% fibrosis in *CHIP*^−/−^ mice [13]), these data suggest that fenofibrate causes some degree of pathophysiological cardiac remodeling in the absence of CHIP and hemodynamic stress.

### 3.6. The Effect of Fenofibrate on Mitochondrial Ultrastructure in the Cardiac Sarcomere and on the Expression of Metabolic, Autophagy, and Mitophagy Genes

**Figure 6:**
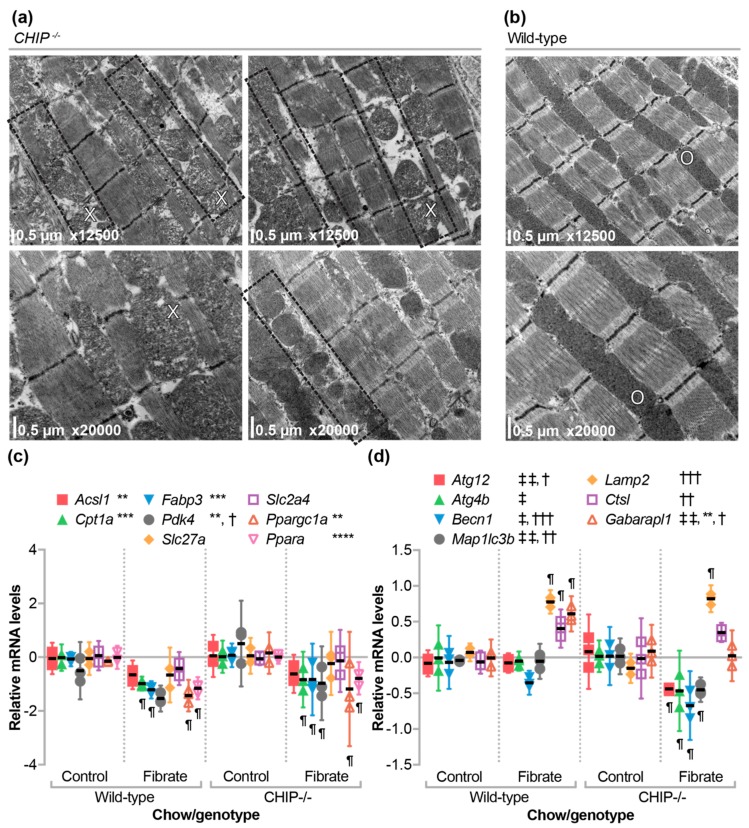
The effect of fenofibrate on mitochondrial ultrastructure and gene expression in the absence of CHIP. Transmission electron micrographs of left ventricle tissue from (**a**) *CHIP*^−/−^ and (**b**) wild-type mice. Expanded sarcoplasm regions are observed throughout *CHIP*^−/−^ hearts (outlined in boxes). Alterations in mitochondria were also observed in *CHIP*^−/−^ hearts (X) and compared to wild-type hearts (O). Quantitative PCR analysis of gene expression of (**c**) metabolic or (**d**) autophagy/mitophagy mRNA in hearts from either wild-type or *CHIP*^−/−^ mice fed control or fenofibrate chow,represented by dot plot and summarized by the mean ± 95% CI, N = 3 animals per genotype per condition: **, ***, **** indicate *p* < 0.01, 0.001, 0.0001 on the main effect of chow, ^†^, ^††^, ^†††^ indicate *p* < 0.05, 0.01, 0.001 on the main effect of genotype, or ^‡^, ^‡‡^ indicate *p* < 0.05, 0.01 the interaction of chow and genotype via two-way ANOVA. ¶ indicates the Tukey’s post-test < 0.05 in comparing fibrate to control conditions within genotypes.

We previously did not observe any structural differences in the sarcomeres of unstressed *CHIP*^−/−^ hearts [13]. However, in hearts from *CHIP*^−/−^ mice treated with fenofibrate, we found various regions with expanded sarcoplasmic reticulum as well as changes in mitochondrial morphology including decreased cristae density (Figure 6a) in comparison with wild-type sarcomeres (Figure 6b). Recycling of protein aggregates and mitochondria occur through the related autophagy and mitophagy pathways. These pathways in the heart are activated by several cardio-protective compounds [42] including fenofibrate [43,44,45]. Moreover, CHIP is known to play an important role in autophagy [12,46,47,48,49,50]. To explore the mechanism behind the phenotype observed in *CHIP*^−/−^ mice, we used qPCR analysis to determine if fenofibrate had differential effects on metabolic or autophagy/mitophagy gene expression in hearts isolated from *CHIP*^−/−^ versus wild-type mice. 

We identified similar patterns of metabolic gene expression in fenofibrate-treated mice, irrespective of CHIP expression (Figure 6c) suggesting that other cellular pathways may be implicated in the phenotypes we observed. In contrast, a different pattern appeared in the analysis of autophagy/mitophagy-related genes (Figure 6d). In wild-type mice, fenofibrate increased the expression of *Lamp2* and *Ctsl*, which are genes that encode enzymes involved in lysosomal function and the formation of the autolysosome [51], and *Gabarapl1*, which is a gene that encodes a phospholipid-interacting protein involved in the later stages of autophagosome maturation [52]. In *CHIP*^−/−^ mice, we also measured a similar increase in *Lamp2* expression (Figure 6d). However, several genes involved in the initiation of autophagosome formation including *Becn1*, *Atg12*, *Atg4b*, and *Map1lc3b* [51] were decreased in *CHIP*^−/−^ mice when treated with fenofibrate (Figure 6d). These data are consistent with the role of CHIP in regulating autophagy-related pathways [12,46,47,48,49,50] and the disruption of regulation is more pronounced in conditions that may activate autophagy/mitophagy such as therapies that include fibrates.

## 4. Discussion

Pressure overload and the subsequent pathological remodeling in the heart is associated with changes in cardiac metabolism [53,54,55]. In the initial compensatory phase of pressure overload, the heart adapts to the increased demand and can maintain cardiac output. However, over time, if the stress is not relieved, this adaptation turns to maladaptation and eventual heart failure. In our mouse model of pressure overload, the initial adaptation is exemplified after one week, since wild-type hearts maintain function in part due to compensatory cardiomyocyte hypertrophy [13]. Deletion of CHIP in mice results in the inability to meet cardiac energy demands during pressure overload, which leads to robust cardiac hypertrophy and impaired cardiac function [13]. This may be reflective of the metabolic inflexibility of *CHIP*^−/−^ mice to pathological stressors. We used metabolomics (Figure 1a) to identify changes that occur during this adaptive phase in both wild-type and *CHIP*^−/−^ mice. We observed an increase in long-chain and medium-chain acylcarnitines (Figure 1b) that was accompanied by an increase in fatty acid oxidation and total oxidation (Figure 1c) as well as increased ATP levels (Figure 1d,e), which is consistent with our previous report [13]. These effects were attenuated in *CHIP*^−/−^ mice (Figure 1b–e), which is consistent with our hypothesis that CHIP is necessary for cardiac metabolic flexibility. We decided to test the effect of stimulating oxidative metabolism pharmacologically using the PPARα agonist, fenofibrate, in *CHIP*^−/−^ mice. Unexpectedly, treating *CHIP*^−/−^ animals with fenofibrate decreased cardiac function (Figure 5a) accompanied with increased cardiac fibrosis (Figure 5b,d). Changes in mitochondrial ultrastructure were observed in *CHIP*^−/−^ mice treated with fenofibrate including a decrease in cristae density (Figure 6a). Fenofibrate can induce changes in hepatic mitochondria in rodents, canines, and humans [56,57]. Mitochondrial impairment is thought to mediate the toxicity of fibrates as well as statins and thiazolidinediones [58]. Therefore, the cardiac effects seen in *CHIP*^−/−^ mice treated with fenofibrate may be a result of impaired mitochondrial function or quality control via mitophagy (Figure 6d).

PPARα target genes in the heart are poorly defined. As such, fenofibrate did not result in increased transcription of several genes involved in fatty acid oxidation in mouse hearts from either genotype (Figure 6c). However, we found that the expression of hepatic genes known to be responsive to PPARα agonists were regulated to a similar extent in wild-type and *CHIP*^−/−^ animals (Figure 2a). This suggests that CHIP expression was not necessary for hepatic PPARα-mediated gene transcription. Likewise, we saw an equivalent increase in liver mass with fenofibrate in mice of either genotype (Figure 2b), which was shown previously in multiple mouse lines [37,38,59]. In contrast, fenofibrate had differential effects on circulating lipids when comparing wild-type and *CHIP*^−/−^ animals. As seen in other mouse models [31,40], fenofibrate increased total, HDL-cholesterol, and LDL-cholesterol levels in wild-type mice, which is a response that was entirely absent in *CHIP*^−/−^ mice (Figure 3b–d). We also found that fenofibrate had deleterious effects on skeletal muscle in *CHIP*^−/−^ mice. These effects include an increase in muscle proteins in the circulation (Figure 4c,d) and a decrease in muscle mass (Figure 4e–g).

Published findings present a mixed picture regarding the effects of fenofibrate on cardiac function in animal models. The majority of studies demonstrate a protective role of fenofibrate on cardiac function by decreasing the degree of cardiac remodeling and fibrosis, which was seen in mouse, rat, and canine models [43,60,61,62,63,64]. In contrast, in mouse models with altered cardiac metabolism, fenofibrate appears to mediate pathophysiological responses. For example, fenofibrate promoted cardiac hypertrophy in mice lacking MuRF1 expression. However, there were no changes in fibrosis, ejection fraction, of fractional shortening [31]. Likewise, fenofibrate treatment increased cardiac hypertrophy and fibrosis along with a decrease in fractional shortening during pressure overload in PPARα deficient mice [65]. These later studies, combined with our results, highlight deleterious consequences of fibrate treatment in the context of altered cardiac metabolism. Clinically, there are reported incidences of fenofibrate-related side effects including liver fibrosis [66] and nephrotoxicity [67]. Moreover, these findings may be particularly relevant in patients with MuRF1 or with CHIP loss-of-function mutations [2,3,68,69] 

Future studies will focus on understanding the metabolic changes accompanying fenofibrate treatment of *CHIP*^−/−^ mice and whether they differ from those in wild-type animals including deleterious effects on cardiac mitochondria. Additionally, defining the integrated stimuli that induces pathological remodeling in *CHIP*^−/−^ hearts will help to elucidate the sensitization caused by the loss of CHIP function. 

## Figures and Tables

**Table 1:** Design of the experiment.

**Genotype**	**No Drug**	**2 w**	**5 w**
Wild-type (N)	8	5	8
*CHIP*^−/−^ (N)	6	4	6
**Analysis**			
Echo	✓	✓	✓
Blood labs	✓		✓
Histology/RNA	✓		✓

**Table 2:** Probes and primers used for qPCR analysis.

Gene	UPL Probe	Sense (5′–3′)	Anti-sense (5′–3′)
*Acox1*	#45	gcgccagtctgaaatcaag	actgctgcgtctgaaaatcc
*Cpt1a*	#109	gctgtcaaagataccgtgagc	tctccctccttcatcagtgg
*Cpt2*	#71	ccaaagaagcagcgatgg	tagagctcaggcagggtga
*Pdk4*	#22	ctgcctgaccgcttagtga	cttctgggctcttctcatgg
*Ucp2*	#2	acagccttctgcactcctg	ggctgggagacgaaacact
*Ppard*	#11	atgggggaccagaacacac	ggaggaattctgggagaggt
*Ppargc1a*	#6	cagtcgcaacatgctcaag	tggggtcatttggtgactct
*Ppara*	#41	cacgcatgtgaaggctgtaa	cagctccgatcacacttgtc
*Acsl1*	#84	cagcctcactgcccttttc	ggttggtggttctctatgcag
*Fabp3*	#56	ctttgtcggtacctggaagc	tggtcatgctagccacctg
*Slc27a*	#1	gacaagctggatcaggcaag	gaggccacagaggctgttc
*Slc2a4*	#5	gacggacactccatctgttg	gccacgatggagacatagc
*Atg12*	SYBR	ggcctcggaacagttgttta	cagcaccgaaatgtctctga
*Atg4b*	SYBR	attgctgtggggtttttctg	aaccccaggattttcagagg
*Becn1*	SYBR	ggccaataagatgggtctga	cactgcctccagtgtcttca
*Ctsl*	SYBR	gtggactgttctcacgctcaag	tccgtccttcgcttcatagg
*Gabarapl1*	SYBR	catcgtggagaaggctccta	atacagctggcccatggtag
*Lamp2*	SYBR	tggctaatggctcagctttc	atgggcacaaggaagttgtc
*Map1lc3b*	SYBR	cgtcctggacaagaccaagt	attgctgtcccgaatgtctc

**Table 3:** Echocardiogram results of study mice. Left ventricular dimension data obtained via conscious echocardiography. When applicable, measurements reported at diastole or systole (d or s respectively), g, grams, bpm, beats per minute, mm, millimeters, IVS, interventricular septum. LVID, left ventricular internal diameter, LVPW, left ventricular posterior wall, LV Vol, left ventricle volume; EF, ejection fraction. Results of two-way ANOVA on the main effects (Geno = genotype, Drug = w of treatment) and the interaction (Inter) are indicated, Tukey’s post-test: *** *p* < 0.001 comparing 2 w vs. 0 w, ^#^, ^##^, and ^###^ indicate *p* < 0.05, 0.01, and 0.001 comparing wild-type vs. *CHIP*^−/−^ at 2 w, ^†^ and ^†††^ *p* < 0.05 and 0.001 comparing 5 w vs. 2 w.

Genotype				Wild-Type			*CHIP* ^−/−^		
Parameter	Effect	*p*	Drug	0	2 w	5 w	0	2 w	5 w
			N	8	5	8	6	4	6
HR (bpm)	Inter	0.915		600.7 ± 23.8	660.3 ± 17.9	640.5 ± 17.8	602.0 ± 28.0	645.3 ± 26.5	625.5 ± 17.5
	Geno	0.085							
	Drug	0.604							
IVS;d (mm)	Inter	0.0004		1.07 ± 0.02	0.99 ± 0.02	1.11 ± 0.03	1.04 ± 0.02	1.3 ± 0.5 ***^,###^	1.04 ± 0.06 ^†††^
	Geno	0.0027							
	Drug	0.146							
LVID;d (mm)	Inter	0.699		3.50 ± 0.16	3.09 ± 0.14	3.35 ± 0.10	3.26 ± 0.14	3.09 ± 0.24	3.3 ± 0.13
	Geno	0.137							
	Drug	0.425							
LVPW;d (mm)	Inter	0.017		1.04 ± 0.03	1.02 ± 0.01	1.09 ± 0.04	1.00 ± 0.042	1.17 ± 0.07	0.99 ± 0.05 ^†^
	Geno	0.143							
	Drug	0.948							
IVS;s (mm)	Inter	0.0029		1.75 ± 0.06	1.69 ± 0.05	1.86 ± 0.05	1.74 ± 0.07	2.02 ± 0.06 ^##^	1.69 ± 0.09 ^†^
	Geno	0.247							
	Drug	0.398							
LVID;s (mm)	Inter	0.263		1.61 ± 0.08	1.39 ± 0.09	1.43 ± 0.05	1.49 ± 0.09	1.51 ± 0.20	1.63 ± 1.11
	Geno	0.572							
	Drug	0.426							
LVPW;s (mm)	Inter	0.473		1.59 ± 0.04	1.57 ± 0.08	1.68 ± 0.07	1.53 ± 0.05	1.53 ± 0.06	1.5 ± 0.05
	Geno	0.782							
	Drug	0.068							
LV Vol;d (µL)	Inter	0.617		51.92 ± 5.57	38.30 ± 4.02	46.04 ± 3.07	43.46 ± 4.40	38.84 ± 7.82	44.86 ± 4.39
	Geno	0.161							
	Drug	0.447							
LV Vol;s (µL)	Inter	0.254		7.46 ± 0.91	5.13 ± 0.88	5.51 ± 0.51	6.16 ± 1.03	6.80 ± 2.42	7.89 ± 1.20
	Geno	0.723							
	Drug	0.329							
LV Mass (mg)	Inter	0.0039		140.18 ± 7.13	109.95 ± 6.87	140.74 ± 8.32	120.86 ± 11.18	150.08 ± 8.21 ^#^	122.31 ± 11.08
	Geno	0.986							
	Drug	0.917							
EF (%)	Inter	0.087		85.42 ± 0.75	86.89 ± 1.0	87.78 ± 1.12	86.05 ± 0.86	83.84 ± 2.40	82.7 ± 1.71
	Geno	0.915							
	Drug	0.021							

## Supplementary Materials

The metabolomics datasets are available online at the Carolina Digital Repository: https://doi.org/10.17615/C6WM1F.
